# 外泌体在恶性肿瘤中的研究进展

**DOI:** 10.3779/j.issn.1009-3419.2020.101.28

**Published:** 2020-08-20

**Authors:** 玉军 郑, 巍 姜, 东妍 陈, 雷 王, 颜君 李, 璐璐 代, 磊 黄, 明吉 王

**Affiliations:** 116001 大连，大连市友谊医院肿瘤内科 Department of Oncology, The Friendship Hospital of Dalian, Dalian 116001, China

**Keywords:** 外泌体, 恶性肿瘤, 肿瘤发生, Exosomes, Malignant tumor, Tumorigenesis

## Abstract

“体液活检”是近些年恶性肿瘤研究的热点，肿瘤细胞外泌体因携带其“母体”肿瘤细胞的部分功能性蛋白及基因，介导信息传递、参与调控机体的生理功能及病理状态在肿瘤的发生发展过程中起着重要作用，并作为肿瘤液态活检的一种新途径。本文旨在对外泌体的结构、生物特性和检测方法在恶性肿瘤发生发展及临床诊断治疗中的作用进行综述。

## Abstract (en)

"Liquid biopsy" have been intensively studied in recent decades. The exosomes carry their maternal partial functional proteins and genes. As a form of intercellular vesicular transport, exosome-mediated intercellular communication participates in a variety of physiological and pathological processes. The information transmission mediated by exosomes is associated with pathophysiological status and plays an important role in the pathogenesis of cancer. Their detection may be beneficial for the diagnosis and treatment of tumor. This article reviews advances in knowledge on the exosome properties, the effect of exosomes on malignant tumorigenesis, and their clinical application. The data will provide a reference for clinical practitioners.

生物体内所有细胞都是通过直接接触或者由细胞因子、趋化因子等分泌蛋白介导进行信息传递。近年来，作为担任这种细胞间信息传递的新因子，细胞外囊泡（extracellular vesicles, EVs）受到了大家的关注。科学家发现，EVs是由细胞分泌的具有磷脂双分子层结构的囊泡，能够通过其携带的生物大分子（蛋白质、RNA、DNA及细胞因子）调节受体细胞的生物学特性，参与细胞的生理和病理过程^[1, 2]^。根据其生物学表型主要分为3种类型：微泡、凋亡小体和外泌体。微泡是由直接向外的细胞膜出芽形成，凋亡小体是由凋亡细胞膜裂解的碎片构成，外泌体则起源于细胞的内吞过程。外泌体在过去30年中对细胞生物学发展具有着革命性贡献。外泌体通过调节细胞与细胞之间的各种生物过程，已成为细胞间通讯的关键介质，由于其独特的结构及生物特性，引起学者们广泛关注。

本文针对外泌体生物结构特点及其在恶性肿瘤发生发展和临床诊断治疗中的作用进行综述。

## 1 外泌体的结构及生物学特性

外泌体是EVs一个重要的亚群，最早可追溯到1983年，由Johnstone等^[3]^在研究网织红细胞分化形成成熟红细胞的过程中提出，并于1987年在超速离心下正式得到这种囊泡，命名为外泌体，是由细胞内多泡体与细胞膜融合而释放到细胞外、直径40 nm-100 nm、密度1.13 g/mL-1.19 g/mL的膜性囊泡状小体^[4]^。形态呈扁形或球形小体，有些为杯状；在体液中以球形为主。肿瘤细胞中过度表达的某些蛋白质在外泌体中也可能会出现，根据不同的细胞来源，外泌体可表达独特的生物蛋白^[5]^，肿瘤细胞外泌体（tumor derived exosomes, Tex）携带大量的mRNA及微小RNA（microRNA, miRNA），在肿瘤发生发展过程中发挥重要作用。外泌体中DNA的相关报道较少。

外泌体最初被认为是一种用来清除细胞中废物的“垃圾袋”，在生理及病理状态下，可以由各种细胞包括免疫细胞、干细胞及肿瘤细胞主动分泌到细胞外，并可将源自“母体”细胞的大量生物分子运载至其他细胞，与肿瘤的发病机制密切相关。其生物学特性包括：第一，由于其体积微小，能够避开单核巨噬细胞对其的吞噬，且能穿过血管壁到细胞外基质，因而广泛存在于人体各种体液中，包括血液、唾液、尿液、脑脊液及胸腹水；其次，由于其具有磷脂双分子层结构，生物学相对稳定，难于降解。外泌体与靶细胞间的信息传递主要通过3条途径实现：受体-配体相互作用；质膜直接融合；吞噬作用内吞。肿瘤细胞较正常细胞分泌更多外泌体，分析Tex成分可以反应其“母体”细胞的分化及功能状态，其大小及表面蛋白具有高度异质性，即使是由同一个细胞分泌，因为不同分化状态，Tex成分亦可不同，这种独特的分子特征用于与不同肿瘤及正常细胞的鉴别。由于外泌体在肿瘤微环境中的细胞间通讯作用，对于肿瘤的发生、发展、转移、免疫逃避及耐药性等均发挥重要作用。

## 2 外泌体的检测

外泌体相关检测目前包括分离纯化、物理特性鉴定及下游功能研究的分析。研究肿瘤患者体液Tex，必须从大的EVs及正常细胞外泌体中分离出来。需要有效的方法捕获Tex并进行定量分析。外泌体的分离纯化技术主要包括超速离心法、密度梯度离心法、色谱法、免疫磁珠法等，在正常细胞或肿瘤细胞培养基上清液、血液、唾液等体液中检测并分离、纯化外泌体。外泌体的物理特性鉴定技术主要包括电镜观察、动态光散射及纳米微粒追踪分析以及可调谐电阻脉冲传感检测技术。外泌体蛋白质的分析技术主要有酶联免疫吸附测定（enzyme linked immunosorbent assay, ELISA）、蛋白印迹和质谱分析等；核酸的分析技术主要有二代测序、基因芯片、实时荧光定量聚合酶链式反应（polymerase chain reaction, PCR）等。随着外泌体参与肿瘤发生发展的报道增多，通过Tex中携带的RNA及蛋白质等分子信息进行诊断和预后判断成为新的研究热点。

## 3 外泌体在肿瘤发生发展中的作用

### 3.1 外泌体和肿瘤微环境

外泌体在调节肿瘤微环境中具有重要作用，包括改变其他细胞的生物学行为、免疫调节和促血管生成作用等。科学家们发现，在很多的肿瘤组织中浸润着正常的免疫细胞，而临床认为慢性炎症会增加发生肿瘤的风险。同时，成纤维细胞和内皮细胞等细胞构成的肿瘤微环境，有助于肿瘤细胞的增殖。此外，对肿瘤异质性的研究发现，肿瘤的异质性包括肿瘤细胞基因异质性、肿瘤微环境的异质性及肿瘤纳米微环境的异质性。Tex具有促肿瘤及抗肿瘤形成的作用，而基质细胞来源的外泌体亦有促进或限制肿瘤进展的作用。

关于肿瘤来源外泌体对内皮细胞（endothelial cells, ECs）作用的大多数研究都集中在促血管生成作用上。例如，来自胶质母细胞瘤的细胞外泌体会刺激ECs的增殖。经肿瘤转染的胰腺细胞系释放的外泌体会刺激ECs小管生成。然而，越来越多的证据表明，肿瘤细胞还通过与ECs的直接作用或通过外泌体破坏了血管的完整性。这种血管完整性的破坏可促进肿瘤细胞在远处扩散和生长。Lin等^[6]^的研究认为，肿瘤细胞来源的外泌体通过触发ECs内质网应激并破坏其完整性来促进肿瘤转移，而这种基于外泌体的作用不依赖于miRNA。

Chen等^[7]^的研究显示：外泌体长链非编码RNA MRPL23-AS1可促进涎腺腺样囊性癌（salivary adnoid cystic carcinoma, SACC）的肺转移，MRPL23-AS1通过与zeste同源物2（Zeste homolog 2, EZH2）增强子形成RNA-蛋白质复合物来正向调节上皮间质转化（epithelial to mesenchymal transition, EMT）。MRPL23-AS1增加EZH2和H3K27me3在E-钙黏蛋白启动子区域的结合。此外，从SACC患者血浆中分离的外泌体中MRPL23-AS1水平较高，外泌体MRPL23-AS1以“外泌体分泌”方式影响肺微血管ECs。富含MRPL23-AS1的外泌体增加了微血管的通透性，并促进了体内SACC的肺转移。

肿瘤组织内乏氧状态下释放的外泌体，刺激周围血管形成，促进肿瘤的生长及转移。恶性黑色素瘤外泌体的miRNA被ECs吸收后，激活JAK-STAT信号通路促进肿瘤血管生成及血管渗透性增加^[8]^。Tex亦可破坏ECs结构的完整性，外泌体miR-105下调内皮细胞的紧密结合蛋白ZO-1，从而提高血管的渗透性，促进肿瘤播散^[9]^。如肺癌细胞外泌体（lung cancer cell-derived exosome, LCC-exosome）中的miR-210通过调节基质细胞中酪氨酸受体激酶A3（Ephrin A3）的含量，促进肿瘤血管的生成^[10]^；而LCC-exosome中的miR-23a则可以通过激活脯氨酰羟化酶（prolylhydroxylase）及抑制紧密结合蛋白ZO-1来促进肺癌的生血管作用^[11]^。

肿瘤相关成纤维细胞（tumor associated fibroblasts, TAFs）是许多肿瘤，包括结肠、胰腺和乳腺肿瘤微环境中最突出的细胞类型，并在肿瘤间质相互作用中发挥关键作用。TAFs形成的内在机制似乎与成纤维细胞、上皮细胞和内皮细胞（通过EMT）、周细胞、骨髓循环成纤维细胞和间充质干细胞分化的肌成纤维细胞有关^[12]^。Tex分泌多种分子诱导成纤维细胞向肌纤维细胞分化，肌成纤维细胞则可以调节肿瘤微环境（如细胞外基质的降解和细胞外透明质酸产量增加）促进癌细胞的侵袭^[13]^。富含转化生长因子β1（transforming growth factor β1, TGFβ1）蛋白的Tex可以将静息状态的成纤维细胞活化形成肌成纤维细胞，从而促进肿瘤血管新生以及肿瘤的生长，而单纯的可溶性TGFβ1虽然可以激活成纤维细胞，但却没有促进血管新生和肿瘤生长的作用^[14]^。反之，TAF源性外泌体亦可促进肿瘤进展，Hu等^[15]^发现TAF外泌体与结直肠癌干细胞的化疗耐药有关，Boelens等^[16]^提示TAF外泌体增加乳腺癌细胞的治疗耐药。

### 3.2 外泌体和肿瘤免疫

由于Tex在肿瘤微环境中的异质性，其免疫活性从抗原呈递到免疫分化^[17, 18]^是复杂动态的，具有双重作用，既可以发挥抗肿瘤免疫的作用，又可帮助肿瘤细胞免疫逃逸。

Wolfers等^[19]^发现，Tex可将携带的肿瘤相关抗原转运至树突细胞（dendritic cells, DCs），DCs摄取抗原后，引发CD8^+^T细胞依赖的抗肿瘤免疫效应，亦可经弹力蛋白HSP70呈递直接激活自然杀伤细胞（natural killer, NK）。此外，Tex转运的miRNA可能像配体一样通过结合Toll样受体（Toll-like receptor, TLR）触发炎症反应^[20]^。尽管上述外泌体的抗肿瘤免疫活性，但其亦通过以下几个方式诱导免疫逃逸：①诱导IL-6表达抑制骨髓DCs祖细胞，阻止DCs的成熟^[21]^；②下调NK细胞亚群（group 2, member D, NKG2D），抑制NK细胞的增殖及功能。Lundholm等^[22]^发现，前列腺癌细胞外泌体可以下调CD8^+^ T细胞和NK细胞的活化受体；③包膜表面含有肿瘤坏死因子超家族配体：肿瘤坏死因子（tumor necrosis factor, TNF）、凋亡相关因子配体（factor related apoptosis ligand, FasL）和凋亡诱导配体（TNF-related apoptosis-inducing ligand receptor, TRAIL）诱导Fas^+^ T细胞凋亡^[23]^；④控制调节及效应T细胞（T regulatory cells, Tregs）的转录，研究^[24]^表明Tex包含多种对免疫细胞有抑制功能的生物分子，同时可以诱导效应T细胞转化为Treg细胞，并促进Treg细胞的扩增。肺癌细胞起源的miRNA-214可以促进Treg细胞的扩增，同时提高Treg细胞内IL-10的水平，而miRNA-214很可能依赖外泌体途径而发挥作用^[25]^。

总之，我们期待外泌体的抗肿瘤活性，深入了解Tex可有助于研发新的有效的抗肿瘤治疗。目前正在进行的临床研究包括应用DC源性外泌体激活肿瘤（如非小细胞肺癌）患者抗肿瘤免疫^[26]^。

### 3.3 外泌体和肿瘤转移

肿瘤在转移过程中，外泌体通过纤维连接蛋白促进细胞骨架重塑、侵袭性伪足形成、迁徙性增加，以利于肿瘤的转移。再通过膜型基质金属蛋白酶（membrane type matrix metalloprotease, MT1-MMP）促进细胞外基质的分解，使细胞外基质及基底膜重塑、血管新生及血管渗透，来促进肿瘤转移。此外，研究^[27]^发现循环血液中的外泌体富含整合素二聚体，可预测乳腺癌和胰腺癌细胞在体内转移的亲器官性，αvβ5整合素决定了对肝脏、α6β4·α6β1整合素决定了对肺部的指向性。2008年，A1-Nedawi等^[28]^首次发现表皮生长因子受体vIII变异体（epithelial growth factor receptor variant III, EGFRvIII）的胶质瘤细胞，可以通过释放富含突变受体蛋白EGFRvIII的外泌体，赋予野生型胶质瘤细胞更强的侵袭能力。富含EGFRvIII的外泌体进入野生型胶质瘤细胞后，激活EGFRvIII调节的下游通路，如VEGF、BCL-X_L_p27，从而增强受体细胞侵袭能力。应翔等^[29, 30]^研究表明，卵巢上皮性肿瘤来源的外泌体在细胞因子信号传导抑制蛋白3/信号传导和转录活化因子3（suppressor of cytokine signaling 3/signal transducer and activator of transcription, SOCS3/STAT3）旁路的参与下，能够富集miR-222-3p并传递给巨噬细胞M2表型的分化，促进卵巢癌细胞的迁移和转移。沈琳^[31]^团队发现，胃癌患者的恶性腹水来源的外泌体能促进肿瘤细胞腹膜扩散，并揭示了这些外泌体的miRNA表达特征。

总之，外泌体通过激活肿瘤相关信号通路、诱导免疫耐受、重塑细胞外基质、增强肿瘤细胞侵袭性和调控肿瘤微环境促进肿瘤的发生发展。

## 4 外泌体在肿瘤中的临床应用

根据“肿瘤细胞会分泌更多的外泌体”、“外泌体中含有肿瘤特异性抗原，及外泌体能运输这些抗原”等的研究，外泌体作为一种新的诊断和治疗的靶标受到广泛的关注。临床上，检测外泌体用于疾病的早期筛查、无创诊断、疗效监控、辅助用药以及作为肿瘤治疗的药物载体。

### 4.1 肿瘤的诊断

Tex作为液体活检广泛用于肿瘤的诊断，包括前列腺癌、胰腺癌、乳腺癌、卵巢癌、脑胶质瘤及恶性黑色素瘤。外泌体中特异性核酸如EGFRvIII，是脑胶质瘤的可靠标志物^[28]^，血清中miR-21可诊断食管晚期鳞状细胞癌^[32]^，血清miR-141水平升高提示转移性前列腺癌^[33]^，分析尿液中miR谱可以诊断膀胱癌^[34]^。Munagala等^[35]^在对小鼠模型研究中发现，肺癌复发的小鼠肿瘤细胞中miRNA-21和miRNA-155水平相较于原发肺癌小鼠明显上调，这两种miRNA在复发肺癌小鼠外泌体中含量也有所增加。Cazzoli等^[36]^在肺腺癌患者的外周血液中发现miR-378、miR-502-5p、miR-629、miR-200b-5p和miR-100水平较肺部肉芽肿和健康患者明显升高。外泌体DNA检测可以揭示肿瘤特异性突变^[37]^，如胰腺导管腺癌（pancreatic ductal adenocarcinoma, PDAC）驱动基因突变。外泌体蛋白亦可用于肿瘤的检测，如肝细胞生长因子受体（hepatocyte growth factor receptor, MET）及磷酸化MET（Tyr1349）在Ⅲ期、Ⅳ期恶性黑色素瘤中比健康人群明显升高^[8]^。孙楠^[38]^发现外泌体中14-3-3ζ蛋白在肺鳞癌诊断中显示出较高的灵敏度和特异度，有望成为肺鳞癌诊断的标志物。2015美国MD安德森癌症中心的研究发现通过大量的胰腺癌患者血清样本分析发现，相比正常人，胰腺癌患者血清中磷脂酰肌醇蛋白聚糖1（glypican-1, GPC1）阳性外泌体显著增高。进一步研究^[39]^发现，GPC1阳性外泌体在早期胰腺癌患者的血清中丰度显著高于正常人群，且具有较高的准确性和敏感性。另外，不同恶性程度的肿瘤细胞分泌的外泌体成分也不同。比如，与低度恶性浆液性卵巢癌细胞来源的外泌体相比，高度恶性浆液性卵巢癌来源的外泌体更能促进肿瘤血管生成^[40]^。有研究^[41]^发现血浆样本中EVs miRNA谱可以鉴别肺磨玻璃结节的良恶性。

### 4.2 外泌体在肿瘤治疗中的作用

基于外泌体在肿瘤微环境及发生发展中的作用，外泌体在抗癌治疗方面具有重大意义。一方面，Tex携带多种肿瘤相关因子，可以作为治疗靶点，通过抑制外泌体的分泌或消除血液循环中的外泌体治疗肿瘤。比如抑制TexmiRs对上皮细胞的信号传导，可以减少肿瘤血管形成及转移^[42]^；另一方面，外泌体可以作为转运各种抗癌药物（如miRNAs和siRNAs等）的载体。外泌体作为药物载体有多个优点：①外泌体来自自体肿瘤细胞，比人工转运载体具有更少的免疫原性；②外泌体有磷脂双分子层，可直接与靶细胞膜融合，从而提高包裹的药物在细胞中的内化；③外泌体具有较小的尺寸，避免被单核吞噬细胞吞噬，并促进其在肿瘤血管中的外渗及在肿瘤组织中的扩散。

另外，外泌体作为抗肿瘤治疗的纳米载体，已经成功运载基因类药物、抗癌药物、抗炎药物及其他类型药物，其中miRNA在基因类药物运输中应用最广泛。miR-122是已知的肝细胞癌（hepatocellular carcinoma, HCC）特异性的抑癌基因^[43]^。HCC对传统的化疗药物有着较高的耐药性，因此，Lou等^[44]^应用间充质干细胞（mesenchymal stem cell, MSC）来源的外泌体运输miR-122，在体内外实验分别证实了miR-122通过改变其靶基因的表达，增加了HCC细胞的自噬和细胞周期的阻滞，抑制了HCC的增殖。相比较于索拉菲尼的单独给药，联合miR-122给药的抑制效果更明显，增加了HCC的化疗敏感性。另外，Pascucci等^[45]^将高浓度的紫杉醇（paclitaxel, PTX）加入到骨髓细胞来源的MSC培养中，MSC摄取并分泌含有PTX的外泌体，通过外泌体将PTX运输至人胰腺癌细胞中，体内、外实验的结果均表明外泌体可有效地运输PTX靶向至肿瘤细胞，并发挥其抗癌作用。

### 4.3 肿瘤外泌体和治疗耐药

研究^[46]^发现从耐药癌细胞中分离出的外泌体，可以将耐药P-糖蛋白转移到药物敏感细胞中，从而传播耐药性。化疗后患者Tex中可见到顺铂及阿霉素^[47, 48]^。另外，外泌体携带特定的miRs调控细胞周期，使敏感的癌细胞发生耐药并且诱导抗凋亡。试验^[49]^发现，LCC来源的外泌体接触顺铂后，可以加速未接触顺铂的LCC对顺铂产生耐药性。进一步的研究^[50]^表明，这种现象的产生与LCC来源的外泌体中的某些miRNAs密切相关。根除这些外泌体可以控制癌细胞的侵袭。此外，纤维母细胞外泌体通过激活STAT1依赖的抗病毒及Notch3信号通路，使乳腺癌细胞对放化疗耐药^[16]^。对多烯紫杉醇或阿霉素耐药的乳腺癌细胞，从其培养液中分离出的外泌体，可以使对化疗药物敏感的人乳腺癌细胞系MCF-7也产生耐药性^[51]^。HER-2过表达乳腺癌患者HER-2阳性外泌体能够抑制曲妥珠单抗的抗增殖活性^[52]^；因此，清除HER-2阳性外泌体，可恢复曲妥珠单抗的抗肿瘤活性^[53]^。清除肿瘤患者血中外泌体亦可减少免疫耐受^[54]^。

## 5 展望

将来的研究聚焦在癌性外泌体的异质性以便更好的了解其扩增克隆，从而进行针对性治疗。血清外泌体RNA及DNA（液体活检），将用于肿瘤的早期诊断，伴随蛋白组学分析将提供恶性肿瘤在体内的演变过程。应用外泌体运载药物提供了一个新的治疗模式。目前对于外泌体的研究正如火如荼，相信在不久的将来我们会了解外泌体在健康及疾病中更多的功能。不可否认，目前外泌体研究的整个流程中成本都很高，从一定程度上限制了它的研究和临床应用，但是相信随着外泌体标准化分离提纯及检测平台的建立，外泌体在肿瘤的诊断和治疗领域将具有非常广阔的应用前景。
